# Oxidative Stress-Driven Autophagy acROSs Onset and Therapeutic Outcome in Hepatocellular Carcinoma

**DOI:** 10.1155/2019/6050123

**Published:** 2019-05-08

**Authors:** Fabio Ciccarone, Serena Castelli, Maria Rosa Ciriolo

**Affiliations:** ^1^Department of Biology, University of Rome “Tor Vergata”, Via della Ricerca Scientifica, 00133 Rome, Italy; ^2^IRCCS San Raffaele Roma, Via di Val Cannuta 247, 00166 Rome, Italy

## Abstract

Reactive oxygen species- (ROS-) mediated autophagy physiologically contributes to management of cell homeostasis in response to mild oxidative stress. Cancer cells typically engage autophagy downstream of ROS signaling derived from hypoxia and starvation, which are harsh environmental conditions that need to be faced for cancer development and progression. Hepatocellular carcinoma (HCC) is a solid tumor for which several environmental risk factors, particularly viral infections and alcohol abuse, have been shown to promote carcinogenesis via augmentation of oxidative stress. In addition, ROS burst in HCC cells frequently takes place after administration of therapeutic compounds that promote apoptotic cell death or even autophagic cell death. The interplay between ROS and autophagy (i) in the disposal of dysfunctional mitochondria via mitophagy, as a tumor suppressor mechanism, or (ii) in the cell survival adaptive response elicited by chemotherapeutic interventions, as a tumor-promoting event, will be depicted in this review in relation to HCC development and progression.

## 1. Oxidative Stress

Reactive oxygen species (ROS) are the by-products of a number of oxygen-centred biochemical reactions and include free radicals, such as superoxide (O_2_^·−^) and hydroxyl radical (OH^·^), as well as nonradical species, such as hydrogen peroxide (H_2_O_2_). Because these species are formed by sequential reduction of oxygen, they can be interconverted either spontaneously or under enzymatic catalysis [1]. Starting from the 70s, when ROS were typically considered dangerous molecules due to the fact that only damaging and irreversible effects on macromolecules were detected as proof of their ability [2], more recently, we moved to a more composite concept of the ROS role. Indeed, these molecules had a Janus-faced behaviour in cell metabolism, strictly related to their concentration. High ROS flux leads to irreversible alteration of target macromolecules contributing to biological damage inside the cells that has been associated with a number of both physiological conditions, such as aging and senescence, as well as pathological states, such as cancer, neurodegeneration, and cell death. On the contrary, low ROS flux is fundamental for cell signaling leading to cell cycle modulation and cell proliferation. Therefore, a balanced redox state is necessary for avoiding cell damage and for fine-tuning protein functions and molecular pathways [1]. The discovery of reversible redox post-translational modifications on protein cysteine residue opened the avenue for specificity of the signaling pathway, because only a small fraction of proteins becomes oxidized when cells are subjected to mild oxidative stress, due to the peculiar characteristic of surrounding amino acids of the target cysteine [3]. Nowadays, we can assert that the ROS-mediated redox signaling is central in the commitment of cell proliferation, stress response, and survival in mild/controlled ROS burst, while a persistent disequilibrium in redox homeostasis culminates in cell death.

Oxidative stress originates from the overproduction of ROS by endogenous (*e.g.*, mitochondria, peroxisomes, and oxygen-handling enzymes) and exogenous sources (*e.g.*, UV, heavy metals, and micronutrients) or by inefficient/exhausted antioxidants [4]. In particular, the endogenous ROS generation can be an inevitable consequence of the oxidative metabolism, by means of the electron transport chain activity inside the mitochondria, or they can represent a weapon through which specialized cells counteract infections; this is the case of transmembrane enzymes belonging to the NOX family of NADPH oxidases actively producing ROS as primary function (Table 1). Cellular antioxidant equipment spans from low molecular weight nonenzymatic scavengers derived from intracellular synthesis or diet to a variety of committed enzymes. Major enzymatic antioxidants include superoxide dismutase (SOD) having the ability to dismutate the superoxide radical, and catalase and glutathione peroxidases (GPxs) able to efficiently eliminate peroxide derivatives. To these, the thioredoxin reductase enzyme should be added for its role in buffering and restoring redox modifications on proteins. The spatial distribution of antioxidant molecules permits to locally counterbalance the effects of ROS. For instance, different isoforms of SOD exist: the Mn-SOD (or SOD2) is localized in the mitochondrial matrix whereas the CuZn-SOD (or SOD1) is sited in the cytosol and in the mitochondrial intermembrane space [1]. The nonenzymatic antioxidant pattern comprises the tripeptide glutathione (GSH), the major soluble antioxidant abundantly present in all cell compartments, and several vitamins such as the lipid-soluble *α*-tocopherol, particularly present in the hydrophobic side of the cell membrane (Table 1). The GSH redox cycle is probably the most important cellular defense system that exists in the cell; GSH not only acts as a ROS scavenger but also functions in the regulation of the intracellular redox state. The system consists of GSH, GPx, and glutathione reductase, and the ability of the cell to regenerate GSH from its oxidized form GSSG is fundamental in buffering oxidative stress [5].

## 2. Regulation of Tumor Biology by Protein Oxidation

**Figure 1:**
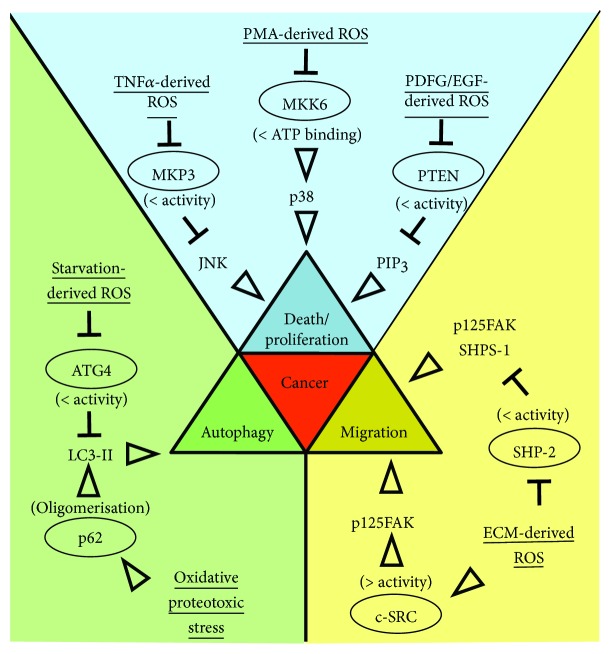
Scheme representing the sources of oxidative stress (underlined) that through direct oxidation affect the function (parenthesis) of key proteins (ellipse) involved in cell death/proliferation, autophagy, and migration/invasiveness, which are pathways commonly altered in cancer. PDGF: platelet-derived growth factor; EGF: epidermal growth factor; PMA: phorbol myristate acetate; TNF*α*: tumor necrosis factor *α*; PIP_3_: phosphatidylinositol 3,4,5 trisphosphate; ECM: extracellular matrix.

ROS levels are typically augmented in many types of cancers. In fact, diverse proliferative signals promote ROS generation as observed for those elicited by growth factor receptors coupled with NADPH oxidases [6, 7]. DNA mutations derived from oxidative DNA damage represent the typical ROS protumorigenic action. Together with 8-oxoguanine (8-oxoG), one of the most common DNA lesions caused by ROS, oxidative damages comprise DNA single-strand or double-strand breaks as well as rearrangement of DNA sequence [8]. However, ROS contribute to cell proliferation even through H_2_O_2_-mediated oxidation of cysteine residues present on a surface-exposed region of oncogenes/tumor suppressors. Several kinases involved in the mitogen-activated protein kinase (MAPK) pathway, which is one of the well-established cell transduction cascade contributing to cell proliferation/survival, have been demonstrated to be regulated in this way. In particular, the modulation of such pathway by oxidation can occur (i) indirectly, as demonstrated for the inhibition of the MAP kinase phosphatase 3 (MKP3) that is a Jun N-terminal kinase (JNK) antagonizing enzyme [9], or (ii) directly, as observed for the inhibition of the mitogen-activated protein kinase kinase 6 (MKK6) that specifically activates the p38 MAPK [10] (Figure 1).

Along with the regulation of cell proliferation-linked pathways that contribute to tumor initiation, ROS are also involved in tumor progression/dissemination facilitating cell motility and metastasis. In this context, a complex succession of redox reactions affects the activity of kinases—*e.g.*, protooncogene c-Src [11] and C-terminal Src kinase (CSK) [12]—and phosphatases—*e.g.*, phosphatase and tensin homolog (PTEN) [13] and Src homology 2 domain-containing phosphatase 2 (SHP-2) [14]—that coordinate the anchorage-independent cell growth downstream of integrin signaling triggered by extracellular matrix binding [15] (Figure 1).

Another process extensively connected with redox signaling in cancer cells is autophagy, which accounts for tumor development/progression in harsh conditions. In the present review, we will circumstantiate the interplay between ROS and autophagy in hepatocellular carcinoma (HCC) focusing on the carcinogenic effects of the wide range of environmental risk factors involved in and on the therapeutic sensitivity/refractoriness of this solid tumor. Before entering the main issue of this review, a brief description of the autophagic process and the ROS-mediated regulation of key players will be provided hereafter.

## 3. (Macro)autophagy

Autophagy typically allows cells to maintain the correct turnover of their component through the degradation of old organelles and proteins recovering energy and macromolecular precursors. Because of its intrinsic role of the recycling pathway, autophagy can regulate physiological functions in which cellular components have to be degraded, building blocks have to be formed, and the cell has to response to stress. The main biological effects inside the cell include differentiation, response to starvation, quality control mechanism through elimination of damaged proteins and organelles, and antimicrobial activity through elimination of bacteria or viruses. Consistently, a lot of different stimuli can activate the autophagic mechanism, demonstrating the complicated nature of this pathway [16].

When we talk about autophagy in this review, we consider the so-called “macroautophagy” which culminates with the fusion of mature autophagosome (the vesicle that contains the components that will be degraded) with lysosome to degrade its content by acidic hydrolases. Other mechanisms include “microautophagy,” in which lysosome directly wraps around its cargo to eliminate it, and “chaperone-mediated autophagy,” in which the binding between a chaperone protein and a target protein forms a complex that is recognized by LAMP2A (lysosomal-associated membrane protein 2) allowing the translocation of target protein into the lysosome [17].

The first step of (macro)autophagy is the formation of phagophore, a membrane structure that wraps parts of the cytoplasm, thanks to the interventions of a complex containing autophagy-related proteins (ATGs). During nutrient deprivation, the canonical autophagic stimulus, the target of rapamycin kinase complex I (TORC1) has a crucial role; according to the current hypothesis, TORC1 is able to sense directly the flux of extracellular amino acids from outside to inside the cell [18]. In starvation condition, the inhibition of the mammalian target of rapamycin (mTOR) allows the activation of uncoordinated-51-like kinases 1 and 2 (ULK1 and ULK2), which together with ATG proteins form the complex that localizes on the phagophore to induce the autophagosome nucleation [19, 20]. Alternatively, the formation of double-membrane structure is induced via Beclin 1 forming distinct phosphatidylinositol 3-kinase complexes [20, 21]. The elongation of phagophore, principally driven by ATG9, determines the formation of a double-membrane vesicle creating the autophagosome, which then fuses with lysosome [20]. The introduction of target protein inside the autophagosome is generally mediated by binding with the light chain 3-II (LC3-II) which localizes at autophagosome surface through phosphatidylethanolamine (PE) post-translational modification [16, 22]. The process of autophagy is selective in principle as many adaptor proteins allow LC3-II to recognize specific targets; among those, p62/sequestrosome 1 (SQSTRM1) has been characterized as the selective mediator of ubiquitinated protein degradation via autophagy [16, 23].

## 4. ROS-Mediated Regulation of Autophagic Flux

Aberrant increase of the endogenous or exogenous source of ROS can induce macromolecule damage associated with oxidative stress that needs to be efficiently managed. In the perspective of cellular homeostasis, autophagy is a crucial response to oxidative stress, and there are many ways through which ROS can activate autophagy. A direct regulation of autophagic machinery is exemplified by H_2_O_2_-mediated oxidation of (i) ATG4 that becomes inhibited and cannot delipidate LC3 promoting its association with the autophagosomes [24] and (ii) p62 that undergoes oligomerization boosting autophagosome biogenesis and autophagic flux [25] (Figure 1).

In addition, ROS are able to influence the signaling pathways involved in autophagy regulation at different levels. For instance, AMP-activated protein kinase (AMPK) is sensitive to oxidative stress both directly or indirectly with repercussion on autophagy induction [26–28]. Indeed, the activation of AMPK leads to the inhibition of TORC1 promoting autophagy. In this regard, the activation of AMPK-dependent autophagy triggered by starvation is mediated by mitochondrial ROS burst [29]. On the other side, ROS-dependent activation of the MAPK14/p38 during starvation is necessary for restraining autophagy activation in cancer cells preserving cell viability in stress conditions [30].

Along with nutrient deprivation, another stressful condition that involves autophagy as an adaptive survival mechanism in hostile environment is hypoxia. Strikingly, although in low oxygen tension conditions, increase of ROS has been extensively documented upon hypoxia [31] and consequences on autophagy induction have been shown. Hypoxia-inducible factors (HIFs), the master regulators of hypoxia response, are indeed able to promote transcription of autophagy key proteins, including BCL2 interacting protein 3 (BNIP3) and NIP-like protein X (NIX) expression [32], which positively regulate autophagy through Beclin 1 activation in oxygen-deprived condition or after mitochondrial ROS generation.

## 5. HCC Risk Factors and ROS

HCC accounts for about 90% of all primary liver cancers worldwide, and due to a high rate of recurrence after resection and a poor response to conservative therapy, it has a very poor prognosis. Apart from genetic predisposition, a large number of environmental and lifestyle risk factors have been documented for HCC, primarily cirrhosis and hepatitis B virus (HBV)/hepatitis C virus (HCV) infections. Among others, nonalcoholic steatohepatitis (NASH), alcohol abuse, metabolic syndrome, and aflatoxin B exposure have to be mentioned [33].

For most of these risk factors, augmentation of oxidative stress has been reported as an accepted mechanism contributing to hepatocarcinogenesis. The X protein (HBx) codified by HBV genome has been associated with increased ROS production in mitochondria where it is associated with the outer membrane affecting human voltage-dependent anion-selective channel isoform 3 (hVDAC3) with consequent mitochondrial depolarization [34–36]. In line with this, the C-terminal region of HBx was shown to be crucial for the formation of oxidative DNA damage at mitochondrial level in terms of 8-oxoG with no evidence of nuclear DNA damage [37]. Increased levels of 8-oxoG were analogously observed in human hepatoma cells infected with HCV *in vitro* [38], as well as *in vivo* in livers of transgenic mice expressing HCV core protein [39]. HCV-mediated ROS production also occurs via alteration of mitochondrial functionality including inhibition of the electron transport chain [40, 41], altered transmembrane potential [39], and endoplasmic reticulum- (ER-) mitochondrial calcium mobilization [42, 43]. Alternative mechanisms leading to augmented ROS production by HCV infection involve the upregulation of NADPH oxidases 1 and 4 subunits via the transforming growth factor *β*1 (TGF-*β*1) signaling or induction of cytochrome P450 2E1 (CYP2E1), which is a cell detoxification system producing different ROS species [44]. Imbalance in oxidative stress was also demonstrated during alcohol abuse which promotes ROS by xanthine oxidase, establishing hypoxic areas in the liver of rodents and humans [45], or by augmented activity of CYP2E1 entailed for ethanol catabolism [46, 47]. It is very interesting to note that no evidence of exacerbated ROS generation is ascribable to augmented activity of fatty acid oxidation and thus electron transport chain flux in lipid-rich condition typical of obesity- and NASH-driven HCC. In fact, analyses of the liver from patients with NASH have only revealed mutations or decreased levels of electron transport chain complexes [48, 49] and ROS generation has been hypothetically associated with CYP2E1 activity, iron accumulation, and ER stress [50]. This may imply that when ROS derive from physiological sources (e.g., lipid beta-oxidation), cells with a high level of metabolic competence like hepatocytes induce homeostatic pathways for managing ROS burst. In support of this, fatty acids liberated by the rate-limiting enzyme of triacylglycerols are able to activate the signaling of nuclear receptors, such as peroxisome proliferator-activated receptors (PPAR), affecting antioxidant response and metabolic adaptations with implications also in HCC development [51, 52].

Apart from genetic aberrations due to direct formation of 8-oxoG on DNA or to lipid peroxidation that induces the promutagenic DNA adduct cyclic *γ*-hydroxy-1, N2-propanodeoxyguanosine [53], oxidative stress also contributes to hepatocarcinogenesis via epigenetic mechanisms. Locus-specific epigenetic changes occurring in HCC cells include the hypermethylation of the E-cadherin promoter by H_2_O_2_ treatment [54] and the hypermethylation of the suppressor of cytokine signaling 3 (SOCS3) due to HBV-induced mitochondrial ROS accumulation [55]. In both these examples, the oncogene Snail is actively involved recruiting the repressive epigenetic enzymes DNA methyltransferase 1 (DNMT1) and histone deacetylase 1 (HDAC1).

## 6. Autophagic Response to Oxidative Stress during HCC Onset

The pivotal tumor suppressor mechanism exploited by autophagy for buffering dangerous ROS production is the removal of damaged mitochondria by mitophagy. This selective engulfment of mitochondrial cargo engages a number of adaptor proteins, such as BNIP3 and NIX, in combination with E3 ubiquitin ligases that operate when localized at mitochondria, such as Parkin and Mitochondrial E3 Ubiquitin Protein Ligase 1 (Mul1). In particular, Parkin dampens HCC development as demonstrated by the proliferative phenotype of hepatocytes and the development of macroscopic hepatic tumors in Parkin knockout mice [56]. The activation of Parkin-dependent mitophagy was also documented in regard of ROS-mediated hepatocarcinogenic effect of ethanol. Accordingly, alcohol induced extensive mitochondrial damage and oxidative stress in Parkin knockout mouse livers, which exhibited decreased mitophagy and mitochondrial respiration [57, 58]. Moreover, relocalization of Parkin at dysfunctional mitochondria largely coincides with accumulated 8-oxoG in the liver from ethanol-treated rats [59]. A cytoprotective role of autophagy and mitochondrial disposal was also described in response to CYP2E1-dependent oxidative stress during chronic ethanol-induced liver injury [60]. The same outcome was inferred after treatment with agents able to stimulate CYP2E1-dependent toxicity, such as polyunsaturated fatty acid, arachidonic acid, and buthionine sulfoximine, in combination with autophagy modulators [61].

On the other hand, ROS-mediated autophagy has been linked with survival mechanisms of HCC to stressful conditions. Autophagic flux and ROS are both increased during ischemia/reperfusion injury, one of the major complications of liver resection, favoring proliferation and survival of HCC cells [62]. In addition, elevated ROS production was associated with the activation of AKT, which induces a survival-promoting autophagy sustaining p53 degradation and NF-*κ*B expression in HCC [63].

Based on this complexity, a univocal functional outcome of the interplay between ROS and autophagy in HCC tumorigenesis cannot be predicted. Moreover, autophagy intersects many of the signaling pathways that are genetically altered (i.e., PI3K/Akt/mTOR, Ras/Raf/MAP kinase, and Wnt-*β*-catenin pathways), most of which are triggered by ROS. It is also to be noted that autophagy is directly involved in the modulation of oxidative stress response via stabilization of Nrf2, the master regulator of antioxidant pathways, by p62-mediated autophagic degradation of the Nrf2 inhibitor Keap1. However, a persistent p62-mediated stabilization of Nrf2 under stressful conditions may overcome the gatekeeper function of autophagy in the liver activating Nrf2-mediated reprogramming of metabolism, stress response, and cell cycle associated with hepatocarcinogenesis [64, 65]. Along with this, a detrimental connection between autophagy and Nrf2 has been also demonstrated in ATG5 liver-specific knockout mice, which spontaneously develop inflammation, fibrosis, and tumorigenesis, but this phenomenon is abolished in the absence of Nrf2 [66]. These examples further highlight how the complicated liaison between ROS signaling and autophagy can contribute to cancer development/progression when it is affected by genetic and chronic environmental insults.

## 7. Autophagic Response to Oxidative Stress during HCC Therapeutic Intervention

Due to molecular heterogeneity of HCC and resistance to common chemotherapy, the most radical curative approaches are therapeutic surgery and liver transplantation, when applicable. Systemic therapy is generally exploited when terminal HCC occurs, and sorafenib is the only standard treatment available [33]. Sorafenib is a multikinase inhibitor impinging on MAPK/ERK-mediated cell proliferation and VEGF-driven angiogenesis thus targeting both tumor cells and endothelial cells [67]. Apoptosis has been classically considered as the major cytotoxic effect of sorafenib *in vitro* and in animal models [68], but typical signs of apoptotic cell death were not frequently observed in HCC patients treated with sorafenib, till to be considered as a weak proapoptotic molecule as a single agent [69]. This is in line with the survival of only two-three months observed for advanced HCC patients cured with sorafenib and the need of developing new effective interventions. In this regard, the research of a combinatorial therapeutic approach for enhancing sorafenib efficacy in HCC has been currently intensified based on the fact that sorafenib elicits a plethora of secondary mechanisms, including both oxidative stress and autophagy. Mitochondria-dependent ROS production accounts for cytostatic and cytotoxic effects of sorafenib in a dose-dependent manner [70, 71]. On the other hand, autophagy induction by sorafenib is largely exploited for adaptive survival response and is triggered by several cues, such as ER stress [72], mTORC1 inhibition [73], or Beclin 1 release from inhibitory factors [74]. However, autophagy seems also to improve the lethality of sorafenib against HCC cells promoting apoptosis, suggesting that individual HCC cells may activate distinct autophagy signaling pathways that allow them to respond differently to chemotherapeutic treatments [75]. Even though oxidative stress and autophagy are concomitant events linked to sorafenib, no mutual relationship has been highlighted so far. Such connection has been instead largely described for alternative chemotherapeutic agents that have been characterized in preclinical models of HCC. Oxaliplatin treatment induces proapoptotic effects via ROS generation, and hindering autophagy exacerbates that phenotype [76]. Autophagy inhibitors also foster apoptotic cell death triggered by bevacizumab in the presence of ROS derived from starvation or hypoxia [77]. The occurrence of apoptotic cell death after salinomycin, capsaicin, propyl gallate, or licochalcone A treatment was instead dependent on a direct impact of these molecules on the autophagic response to oxidative stress [78–81] (Table 2).

Notably, a number of papers have also clarified that the induction of ROS-mediated autophagic flux can culminate in HCC cytotoxicity via autophagic cell death (ACD). This mechanism was proposed during hyperthermia-dependent radiotherapy sensitization [82] and demonstrated after treatment with chemotherapeutic compounds. The administration of OSU-03012, a synthetic compound acting as a 3-phosphoinositide-dependent kinase 1 (PDK1) inhibitor, elicits ACD in HCC cells differently from other tumor types where it triggers apoptosis. Moreover, in HCC, this outcome is independent of PDK1 but connected with ROS-mediated autophagy induction [83]. Analogously, the adrenal steroid hormone dehydroepiandrosterone (DHEA) induces ACD only in some cellular contexts [84, 85], including HCC, where it acts independently of the typical inhibition of the glucose-6-phosphate dehydrogenase (G6PDH) triggering ROS burst and oxidative stress [86]. The dosage of the alkaloid tetrandrine in HCC cells was instead demonstrated to tip the balance in favor of apoptosis or ACD at high and low concentrations, respectively. In this context, ACD was triggered by ROS-mediated activation of the ERK signaling pathway and overexpression of ATG7 [87]. In many other cases, such as after treatment with the alkaloid tetramethylpyrazine or with the novel manganese (II) compound Adpa-Mn, induction of ROS-dependent autophagy and apoptosis is a concomitant event necessary for therapeutic success [88, 89] (Table 2).

The complex scenario described here can be justified by the fact that several stimuli and pathways, including ROS, are shared by autophagy and apoptosis, and a number of mutually regulatory mechanisms exist. In this context, Beclin 1 plays a central role at the molecular level. For instance, during sustained exposure to apoptotic insults, Beclin 1 is a target of caspase proteolytic activity that generates fragments able to inhibit autophagy but to stimulate apoptosis. Moreover, Beclin 1 is inhibited by the interaction with the antiapoptotic protein Bcl-2 whereas Beclin 1-mediated autophagy prevents apoptosis by degrading the active caspase-8 [90].

## 8. Conclusion

**Figure 2:**
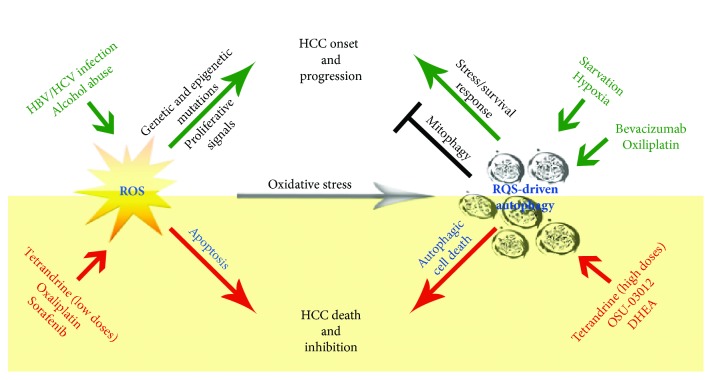
Interplay between ROS and autophagy in HCC as consequence of risk factors, environmental stress conditions, and therapeutic treatments.

Oxidative stress is a critical event linked to hepatocarcinogenesis and virtually associated with all the wide range of environmental risk factors contributing to HCC onset (Figure 2). In general, increased levels of ROS are responsible for genetic/epigenetic mutations and proliferative signals, but a failure in the counterbalance of ROS by antioxidants predisposes to cell death. Autophagy is also a double-edged sword in the context of HCC onset/progression. Autophagy acts as a tumor suppressor mechanism in the liver avoiding proteotoxicity [91] and aberrant mitochondrial ROS production via mitophagy [56] (Figure 2), but it also accounts for tumor adaptation to stressful conditions, such as starvation and hypoxia, mostly in the inner core of the tumor [62, 63]. The interplay between ROS and autophagy has a strong impact on therapeutic outcomes. Several prooxidant molecules tested in HCC were shown to induce simultaneously apoptotic cell death and autophagy. However, the latter can act as a prosurvival response typically associated with drug resistance or as an alternative/combined cytotoxic mechanism in terms of autophagic cell death (Figure 2). This definitively indicates that targeting autophagy in HCC is a complex approach that needs to be carefully evaluated for the success of a therapeutic strategy based on ROS-generating drugs.

## Figures and Tables

**Table 1:** Main ROS sources/antioxidants and their localization.

Endogenous ROS sources	Main localization	Antioxidants	Main localization
NADPH oxidase	Plasma membrane	Glutathione	Ubiquitous
Respiratory complexes I and III	Mitochondrion	*α*-Tocopherol	Plasma membrane
CYP450	Endoplasmic reticulum	Superoxide dismutase	Mitochondrion/cytosol
Xanthine oxidase	Cytosol/peroxisome	Catalase	Peroxisome
Peroxisomal oxidases	Peroxisome	Thioredoxin	Nucleus/mitochondrion
Lipoxygenase/cyclooxygenase	Cytosol	Glutathione peroxidase	Cytosol/mitochondrion/plasma membrane

**Table 2:** Molecules tested in HCC cell lines and impinging on ROS/autophagy crosstalk.

Drug	Effect	Role of ROS	Source of oxidative stress	Ref.
Sorafenib	Apoptosis and prosurvival autophagy	Dose-dependent cytostatic and cytotoxic effects; apoptotic cell death	Mitochondrial ROS and GSH depletion	[70, 71]
Oxaliplatin	Apoptosis and prosurvival autophagy	Enhanced apoptotic cell death upon autophagy inhibition	Inhibition of thioredoxin reductase	[76, 92]
Salinomycin	Apoptosis and inhibition of autophagy	Contribution to apoptosis activation	Accumulation of dysfunctional mitochondria due to impaired autophagic flux	[79]
Capsaicin	Apoptosis and induction of cytoprotective STAT3-dependent autophagy	Phosphorylation of STAT3 and activation of autophagy	Inhibition of mitochondrial complexes I and III; reduction of antioxidants (results obtained in pancreatic cancer cells)	[80, 93]
Licochalcone A	Induction of apoptosis and prosurvival ULK1/ATG13-mediated autophagy	Activation of autophagic flux	Suppression of the GSH generation and formation of superoxide	[78]
Bevacizumab	Antiangiogenic effect and induction of prosurvival autophagy	Enhancement of metabolic stress-induced oxidative damage and cytotoxicity	Indirectly obtained by metabolic stress such as starvation and hypoxia	[77]
OSU-03012	Autophagic cell death (ACD)	Activation of autophagic flux	Unknown. Mitochondrial superoxide production was demonstrated for the analogue Celecoxib	[83, 94]
DHEA	ACD	No involvement in autophagic commitment	Decrease of GSH/GSSG ratio and impaired pentose phosphate pathway	[86]
Tetrandrine	Apoptosis (high concentrations) and ACD (low concentrations)	Activation of ERK-mediated autophagic flux	Mitochondrial dysfunction	[87]
Adpa-Mn	Apoptosis and ACD	Induction of both apoptosis and autophagy	Mitochondrial dysfunction	[88]
